# The clinical outcomes of surgical treatment for chronic ankle instability by anatomical reconstruction of the anterior talofibular ligament with autologous half-bundle peroneal longus tendon: A retrospective study

**DOI:** 10.3389/fsurg.2022.926825

**Published:** 2022-08-05

**Authors:** Yeqiang Luo, Shanghui Lin, Lingchuang Kong, Yan Jin, Renkai Wang, Ying Zhang, Baofeng Li, Bei Chen

**Affiliations:** ^1^ Department of Orthopedics, General Hospital of Southern Theater Command, The first School of Clinical Medicine, Southern Medical University, Guangzhou, China; ^2^ Department of Radiation Oncology, Nanfang Hospital, Southern Medical University, Guangzhou, China

**Keywords:** anatomical reconstruction, anterior talofibular ligament, autologous tendon, chronic ankle instability, ligament repair, peroneal longus tendon

## Abstract

The anterior talofibular ligament is the weakest and most vulnerable lateral ligament to be injured, and it can replace anatomical position through anatomical reconstruction. The purpose of this study is to evaluate clinical outcomes after an autologous half-bundle peroneus longus tendon anatomical reconstruction. We conducted a retrospective analysis by enrolling 34 patients [22 male and 12 female, median age 21 (range 19–26) years] with anterior talofibular ligament injury from January 2018 to March 2020. All patients underwent a ligament anatomical reconstruction operation with autologous half-bundle peroneus longus tendon and followed up with an average time of 16.21 ± 3.20 (range 12–24) months, with no loss of patients to follow-up during the study period. The American Orthopedic Foot, Ankle Society Score (AOFAS), Visual Analogue Score (VAS), and Anterior Tibiotalar Translation were used to assess the curative effect. All the indexes were compared between the preoperative and at the final follow-up to discover the related statistical differences. The AOFAS score improved significantly from an average preoperative score of 56.91 ± 3.79 to 94.12 ± 2.51 at the final followed-up (*p* < 0.001). Meanwhile, the pre-operation VAS pain score decreased from 5.94 ± 1.32 to 1.71 ± 0.87 (*p* < 0.001). Additionally, the Anterior tibiotalar translation decreased from 16.40 ± 1.85 to 5.20 ± 0.57 mm at the final followed-up (*p* < 0.001). The anterior drawer test was negative for all patients after the operation. Considering the outcomes, we concluded that anatomical reconstruction of the anterior talofibular ligament with autologous half-bundle peroneal longus tendon was a proper and safe procedure for chronic lateral ankle instability, and it had good clinical results and minimal complications.

## Introduction

Ankle sprains are a clinically common sports injury disease. According to the literature, 80% of all ankle sprains involved the lateral ligaments of the ankle (1). The anterior talofibular ligament is the weakest and most vulnerable lateral ligament to be injured because of anatomical differences in the lateral ligaments. Although conservative treatment for most acute lateral ankle ligament injuries is effective, some of them will continue to develop chronic ankle instability which eventually leads to traumatic arthritis (2). Over the past few decades, there have been many types of surgery for chronic ankle instability, including ligament repair, anatomical reconstruction, non-anatomical reconstruction, and arthroscopic techniques (3–6). Non-anatomical reconstruction surgery uses local tendon grafts to repair ligaments, without the need to repair ligament remnants. However, many studies have shown that subtalar joint stiffness can ensue from non-anatomical reconstruction (7–9). Therefore, arthroscopic and anatomical repairs and reconstructions have recently become a popular treatment for injuries of the anterior talofibular ligament. Direct ligament repair, known as the Brostrom technique, remains the historic benchmark (4). Anatomical repair is based on the anatomical position of the ligament to recover the normal anatomical structure and joint biomechanics of the ligament, and it has become increasingly popular. We were interested in determining if using an autologous half bundle peroneus longus tendon would work best to reconstruct the anterior talofibular ligament. We hypothesized that this operation had good clinical outcomes and a low risk of complications. We compared the American Orthopedic Foot, Ankle Society Score (AOFAS), Visual Analogue Score (VAS), and Anterior Tibiotalar Translation between the preoperative and final followed-up to evaluate clinical outcomes after an autologous half bundle peroneus longus tendon anatomical reconstruction.

## Patients and methods

### Patient selection

The ethical committee of the General Hospital of Southern Theater Command of PLA approved the study. In this study, we retrospectively analyzed 34 patients [22 male and 12 female, median age 21 (range 19–26) years] who underwent a ligament anatomical reconstruction operation using autologous half-bundle peroneal longus tendon for anterior talofibular ligament injury in our hospital from January 2018 to March 2020. All patients were informed preoperatively of the procedure's risks, advantages, and disadvantages, and signed an informed consent form. All procedures performed in studies involving human participants were following the ethical standards of the institutional and national research committee and with the 1964 Helsinki Declaration and its later amendments or comparable ethical standards. For this type of study, formal consent is not required.

Inclusion criteria included all patients who had pain and discomfort in the lateral ankle joint, and the ankle joint anterior drawer test was positive. All patients had a previous history of repeated varus ankle sprains more than 3 times, and 6 months of conservative treatment without improvement. The ankle stress plain radiographs showed anterior tibiotalar translation, and the MRI showed the anterior fibular ligament's disorganized or absent structure. These patients had no foot or ankle fractures and had not received any foot or ankle surgery.

Exclusion criteria included acute injury to the lateral ankle ligament and patients with the chronic consumptive disease or medical disease who could not tolerate the surgery. Patients with severe ankle osteoarthritis or a history of foot and ankle fractures or surgery were also excluded from the study. The presence of concomitant ligament injury was also an exclusion criterion.

### Operative technique

**Figure 1:**
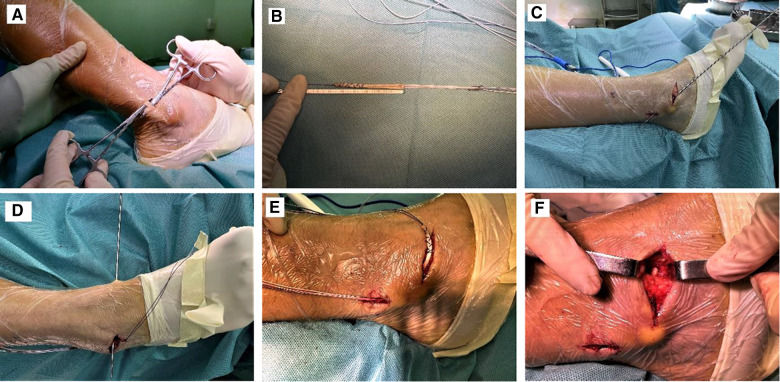
The surgical procedure of anatomical reconstruction of the anterior talofibular ligament with autologous half-bundle peroneal longus tendon. (**A,B**) The autologous half-bundle peroneal longus tendon preparation. (**C**) Fibular tunnel preparation. (**D**) Talus tunnel preparation. (**E,F**) The autologous half-bundle peroneal longus tendon implantation.

All patients were operated on by the senior author (B.F.L.) and the first assistant (L.C.K.). The patient was placed in a supine position, combined spinal-epidural anesthesia. After satisfactory anesthesia, a tourniquet was placed on the root of the thigh of the affected limb. A drape was disinfected routinely after blood evacuation. An arc-shaped incision of 3–4 cm was made below the tip of the lateral malleolus. The subcutaneous tissue and fascia were isolated, and the sural nerve was carefully protected. During the operation, the ankle anterior drawer test and varus stress test were performed again to check the ankle stability and explore the ligament damage, which confirmed that the anterior talofibular ligament could not be anatomically repaired, requiring reconstructive surgery. Graft preparation: We made a longitudinal incision of about 1 cm which was made along the posterior side of the lateral malleolus, and the peroneus longus tendon was fully exposed in the surgical field. the peroneus longus tendon was bisected into two bundles with a hemostat, one was large and the other is small. We chose the small bundle, which was about a third of the total tendon. The tendon was extracted with a tendon extractor. The residual muscle was removed from the tendon and the tendon was folded into double strands with 6 cm in length and 4.5 mm in diameter. The folded end was fixed by the adjustable Arthrex Endobutton loop (Arthrex Endobutton loop ®; Rui Shi Medical Instrument Co, Shanghai, China). Then, using a suture to suture the ends of the autologous half-bundle peroneus longus tendon (Figures 1A,B). The tendon graft was pre-loaded 15 lb for ten minutes. Fibular tunnel and talus tunnel preparation: A 3.0 mm Kirschner wire was drilled from the end of the anterior talofibular ligament toward the posterolateral border of the fibular, and using a 4.5-mm cannulated drill system to prepare for the fibular bone tunnel. The guidewire was placed at a 30 angle from the fibular shaft and 5 to 7 mm from the articular surface of the distal fibula (Figure 1C). The ATFL's talus attachment was debrided, and a tunnel was made in the footprint of the talar attachment. The nosed guide needle was drilled vertically from the outside to the inside of the talus along the guide needle with 4.5 mm in diameter and with a depth of 20 mm (Figure 1D). Under the guidance of the needle, two sutures were respectively passed through the bone tunnels as a traction suture. The tendon was gradually pulled into the fibular tunnel until the adjustable Arthrex Endobutton loop reached the exit of the fibular tunnel, and then the tendon was reverse-pulled until the loop was close to the posterior margin of the fibula. The ankle joint was fixed in a neutral position, and the other end of the tendon was pulled into the talus tunnel and fixed the tendon with a 4.5 * 20 mm absorbable interference screw (MILAGRO ADVANCE, Puits Godet 20 Neuchatel, CH 2000, Switzerland). After the ankle flexion and extension activity were good, the tail of the braided tendon was fixed by an extrusion screw of the appropriate size (Figures 1E,F). Checked ankle range of motion and stability, layered by layered suture closure incision.

### Rehabilitation

The ankle joint was fixed in a neutral position with a short leg cast for 4 weeks after surgery. Patients were allowed to walk non-weight- bearing with double crutches. In the early stage, the toes were moved slowly and to the maximum extent possible without causing pain in the ankle joint, and straight leg raising training of both lower extremities could be performed. At 4 weeks postoperatively, the patient should start walking on crutches and perform functional rehabilitation, including muscle strength and balance training. At 6 weeks postoperatively, the patient is allowed to be fully weight-bearing, and the ankle dorsiflexion, plantar flexion, varus and valgus resistance muscle exercises could be performed using yoga elastic bands.

### Evaluation of curative effect

In our study, we used the Visual Analogue Scale (VAS) score to evaluate ankle pain. We used the American Orthopaedic Foot and Ankle Society score (AOFAS) to assess ankle functional recovery, and we followed up to observe the occurrence of complications. The AOFAS score consists of three components: pain (40 points), function (50 points) and alignment (10 points). The total score of 90–100 is considered excellent, 75–89 is good, 50–74 is acceptable, and less than 50 is poor. We also measured the preoperative and postoperative anterior tibiotalar translation to evaluate the outcomes.

### Statistical analysis

Statistical analysis was performed by IBM SPSS Statistics for Windows, Version 21.0(IBM, USA). A normality analysis was performed to continuous variables using the Shapiro-Wilk test. To compare the pre-operative and post-operative indexes, Paired Samples Test was applied for parametric data. The value of *p* < 0.05 was considered statistically significant.

## Results

We enrolled 34 anterior talofibular ligament injury patients treated by the autologous half-bundle peroneal longus tendon anatomical reconstruction and they were subsequently followed up at an average time of 16.21 ± 3.20(range 12–24) months, with no loss of patients to follow-up during the study period. The demographic of all patients was summarized in Table 1. Data are shown as mean with ± standard deviation and (range) unless otherwise indicated. In the final follow-up, there were significant improvements in the anterior drawer test results, American Orthopedic Foot and VAS score, and Tegner active level, as seen in Table 2. The AOFAS score improved significantly from an average preoperative score of 56.91 ± 3.79 to 94.12 ± 2.51 at the final follow-up (*p* < 0.001). Meanwhile, the pre-operation VAS pain score decreased from 5.94 ± 1.32 to 1.71 ± 0.87 (*p* < 0.001). Additionally, the Anterior tibiotalar translation decreased from 16.40 ± 1.85 to 5.20 ± 0.57 mm at the final follow-up (*p* < 0.001). The anterior drawer test was negative in all patients after the operation. 2 patients had a local complication. At the final follow-up, 2(5.88%) patients had local complications. One patient still had recurrent sprains after surgery, and the patient felt pain and swelling in the ankle joint, which could be due to traumatic arthritis caused by repeated ankle sprains. Another patient had pain and swelling in the affected ankle, which may be caused by the friction between the ankle muscle and the adjustable Arthrex Endobutton loop. The complications of all patients were summarized in Table 3.

**Table 1:** Demographic data of all included patients (mean ± standard deviation).

Variable	Data
Age (years)	21.88 ± 2.10(19–26)
Gender	Male *n* = 22/Female *n* = 12
Side	Left *n* = 15/Right *n* = 19
Follow-up (months)	16.21 ± 3.20(range 12–24)

**Table 2:** Results of anatomic ATFL reconstruction with autologous half-bundle peroneus longus tendon for CLAI (mean ± standard deviation).

	Pre-operation	Final follow-up	*p*-value
AOFAS	56.91 ± 3.79	94.12 ± 2.51	*p* < 0.001
VAS	5.94 ± 1.32	1.71 ± 0.87	*p* < 0.001
Anterior tibiotalar translation (mm)	16.40 ± 1.85	5.20 ± 0.57	*p* < 0.001

**Table 3:** Complications of anatomic ATFL reconstruction with autologous half-bundle peroneus longus tendon for CLAI.

Pain	2/34
Ankylosis	0/34
Infection	0/34
Swelling	2/34
Delayed Wound Healing	0/34
Recurrent Sprains or Instability	1/34
Osteoarthritis	1/34

## Discussion

**Figure 2:**
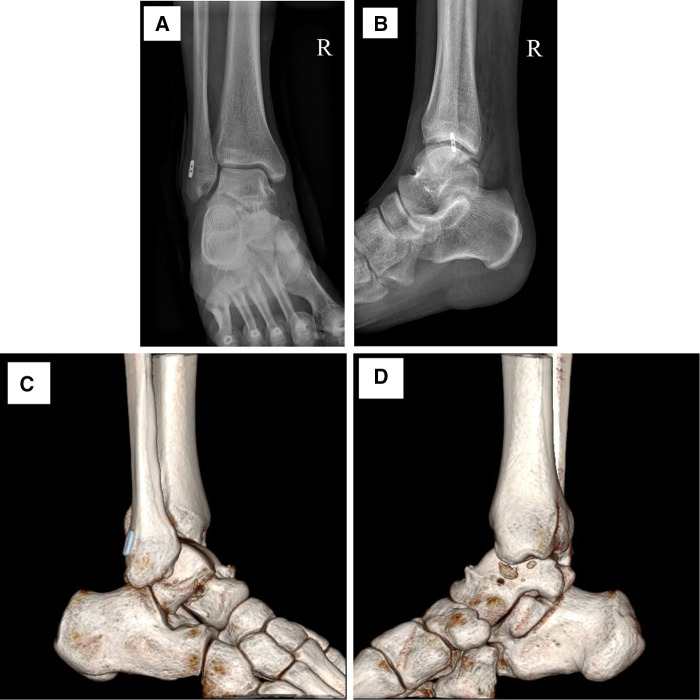
A typical case of the anterior talofibular ligament injury after anatomical reconstruction with autologous half-bundle peroneal longus tendon. (**A**) Post-operative X-ray image in the anterior position. (**B**) Post-operative X-ray image in the lateral position. (**C,D**) Post-operative CT reconstruction image in the sagittal position.

Acute lateral ankle ligament injury is one of the most common problems in foot and ankle medicine (10). If the ligament injury is not timely treated, the patient's foot will be repeatedly inverted during walking, which will eventually go on to chronic ankle instability and wear and tear of the ankle cartilage, which can lead to traumatic arthritis of the ankle joint and affect the patient's daily life. In our study, we rebuilt the anterior talofibular ligament with an autologous half-bundle peroneus longus tendon to increase the stability of the ankle joint. The fibula was fixed with an Endobutton adjustable loop, and the talus was fixed with an Arthrex extrusion screw (Figures 2A–D). We found that the postoperative AOFAS score increased 37.21 points on average compared with that before the operation. The ankle flexion and extension, hindfoot activity, and healthy side were not significantly different, and satisfactory results were obtained. Moreover, during the operation, the method of anterior cruciate ligament reconstruction was adopted. Based on the anatomical operation of the anterior talofibular ligament, the tendon was fixed by Endobutton adjustable loop. The operation was simple, the damage to surrounding tissues was reduced, the operation time was greatly shortened, and the purpose of anatomical reconstruction was achieved. Meanwhile, a small surgical incision is conducive to the early functional exercise of patients.

With the development of medical imaging, there are numerous measures to diagnose ligament injury, including stress radiography, MRI, and ultrasound. Clinical assessment of ankle stability is complicated by the fact that lateral ligament tensions vary with relative degrees of ankle dorsiflexion or plantar flexion (11). The anterior drawer test (ADT) and the talar tilt angle are commonly used in clinical practice to assess lateral ankle ligament injury. The ATFL is an anatomic linkage between the distal fibula and the talus, which limits the sagittal talar translation and axial rotation (12). Phisitkul et al. (13) studied that the ADT is more accurate in diagnosing ATFL injury than standard direct AD testing. Imaging plays a key role in diagnosing ATFL injury. Imaging modalities used to assess lateral ankle ligament stability include stress radiographs, MRI, and ultrasound. Stress radiographs have acted as an objective supplement to physical examination (14). However, studies have shown that the substantial translation and rotation components inherent in any AD maneuver and the TT testing have potential confounding factors for subtalar motion (10). Hoffman et al evaluated the accuracy of stress radiography and concluded that stress radiographs underestimate anterior talar translation and talar tilt angles (14). Morvan et al. (15) found that MRI was sensitive for identifying ATFL abnormality. However, MRI has a high sensitivity but low specificity. As a result, using MRI to recognize additional periarticular pathology rather than as a primary diagnostic tool for CLAI (12). Therefore, when the diagnosis of ankle ligament injury is clear, early surgical treatment should be actively performed.

For an acute injury, conservative treatment is the first choice, and the principle of RICE (Rest, Icing, Compression, Elevation) is often followed in clinical practice. Surgical treatment for patients with an old sprain or residual function instability. As with many advancements in the field of orthopedic surgery, there are very many methods for surgical treatment of anterior talofibular ligament injury in the clinic, and the current prevailing methods are largely simple repair, anatomical repair, and autologous or allogeneic tendon reconstruction. Nonanatomic reconstruction surgery uses local tendon grafts to repair ligaments, without the need to repair ligament remnants. However, many studies have shown that subtalar joint stiffness can ensue from the nonanatomic reconstruction (7–9). Colville et al. (8) reported that Evans, Chrisman-Snook, and Waston-Jones techniques did not restore physiologic ankle range of motion. In recent years, the arthroscopic-Brostrom technique incorporates the inferior extensor retinaculum into the primary construct. It has the advantages of less trauma, no need to sacrifice autologous tendons, and better biomechanical strength, and has been widely recognized. However, some scholars suggested that this operation could cause ligamentous laxity and various hindfoot malalignment. Additionally, it was not suitable for those patients who had fewer ligament stumps and poor surrounding tissue conditions. As a result, although the Brostrom technique is the golden standard, it can only be used when the tissue quality of the elongated ligament is sufficient for repair (16–21). As an alternative to direct ligament repair, anatomical reconstruction using autologous or allogeneic tendons has become increasingly popular. The anatomical reconstruction had similar strength and stiffness to the native ligament and could restore the ankle joint. However, the clinical circumstances that require the use of graft reconstruction remain undetermined. As a result, compared with traditional surgery, anatomical reconstruction of the anterior talofibular ligament with autologous half-bundle peroneal longus tendon, using Endobutton adjustable loop fixation tendon, could make less surgical trauma, helps patients with early rehabilitation, and reduce the damage to the surrounding soft tissue. It was an effective anatomical reconstruction of the anterior talofibular ligament and obtained a satisfactory curative effect.

### Limitations

Firstly, in this study, we used an adjustable Arthrex Endobutton loop, which may increase the patient's financial burden and, owing to individual differences, may cause local complications; Secondly, we did not take into account gender differences, physical activity and postoperative functional rehabilitation training, which is also a limitation of this study. Thirdly, the average follow-up time of patients is short, and the long-term efficacy needs further observation. Moreover, due to anatomical differences in the fibula, it was easy to cause fibula fracture when preparing the fibula tunnel. Finally, the AOFAS score used is not a validated outcome scale for the evaluation of ankle instability, and consequently, some clinical aspects may have been overlooked.

## Conclusion

Anatomical reconstruction of the anterior talofibular ligament with autologous half-bundle peroneal longus tendon was a proper and safe procedure for chronic lateral ankle instability, and it had good clinical results and minimal complications.

## Data Availability

The original contributions presented in the study are included in the article/Supplementary Material, further inquiries can be directed to the corresponding author/s.

## References

[1] Ferran NA Maffulli N. Epidemiology of sprains of the lateral ankle ligament Complex. Foot Ankle Clin. (2006) 11(3):659–62. 10.1016/j.fcl.2006.07.002 16971255

[2] Behrens SB Drakos M Lee BJ Paller D Hoffman E Koruprolu S Biomechanical analysis of brostrom versus brostrom-gould lateral ankle instability repairs. Foot Ankle Int. (2013) 34(4):587–92. 10.1177/1071100713477622 23391625

[3] Brattstrom H. Tenodesis employing the method of watson-jones for the treatment of recurrent subluxation of the ankle. Acta Orthop Scand. (1953) 23(2):132–6. 10.3109/17453675308991205 13138109

[4] Broström L. Sprained ankles. VI. Surgical treatment of “chronic” ligament ruptures. Acta Chir Scand. (1966) 132(5):551–65. PMID: 5339635

[5] Chrisman OD Snook GA. Reconstruction of lateral ligament tears of the ankle. An experimental study and clinical evaluation of seven patients treated by a new modification of the elmslie procedure. J Bone Joint Surg Am. (1969) 51(5):904–12. 10.2106/00004623-196951050-00007 4978936

[6] Gould N Seligson D Gassman J. Early and late repair of lateral ligament of the ankle. Foot Ankle. (1980) 1(2):84–9. 10.1177/107110078000100206 7274903

[7] Bahr R Pena F Shine J Lew WD Tyrdal S Engebretsen L. Biomechanics of ankle ligament reconstruction. An in vitro comparison of the broström repair, watson-jones reconstruction, and a new anatomic reconstruction technique. Am J Sports Med. (1997) 25(4):424–32. 10.1177/036354659702500402 9240973

[8] Colville MR Marder RA Zarins B. Reconstruction of the lateral ankle ligaments. A biomechanical analysis. Am J Sports Med. (1992) 20(5):594–600. 10.1177/036354659202000518 1443330

[9] Hollis JM Blasier RD Flahiff CM Hofmann OE. Biomechanical comparison of reconstruction techniques in simulated lateral ankle ligament injury. Am J Sports Med. (1995) 23(6):678–82. 10.1177/036354659502300607 8600733

[10] Guillo S Bauer T Lee JW Takao M Kong SW Stone JW Consensus in chronic ankle instability: aetiology, assessment, surgical indications and place for arthroscopy. Orthop Traumatol Surg Res. (2013) 99(8 Suppl):S411–9. 10.1016/j.otsr.2013.10.009 24268842

[11] Fujii T Kitaoka HB Watanabe K Luo ZP An KN. Ankle stability in simulated lateral ankle ligament injuries. Foot Ankle Int. (2010) 31(6):531–7. 10.3113/FAI.2010.0531 20557820

[12] Chang SH Morris BL Saengsin J Tourné Y Guillo S Guss D Diagnosis and treatment of chronic lateral ankle instability: review of our biomechanical evidence. J Am Acad Orthop Surg. (2021) 29(1):3–16. 10.5435/JAAOS-D-20-00145 33347006

[13] Phisitkul P Chaichankul C Sripongsai R Prasitdamrong I Tengtrakulcharoen P Suarchawaratana S. Accuracy of anterolateral drawer test in lateral ankle instability: a cadaveric study. Foot Ankle Int. (2009) 30(7):690–5. 10.3113/FAI.2009.0690 19589318

[14] Hoffman E Paller D Koruprolu S Drakos M Behrens SB Crisco JJ Accuracy of plain radiographs versus 3d analysis of ankle stress test. Foot Ankle Int. (2011) 32(10):994–9. 10.3113/FAI.2011.0994 22224329

[15] Morvan A Klouche S Thes A Hardy P Bauer T. Reliability and validity of preoperative mri for surgical decision making in chronic lateral ankle instability. Eur J Orthop Surg Traumatol. (2018) 28(4):713–9. 10.1007/s00590-017-2116-4 29299765

[16] Nery C Raduan F Del Buono A Asaumi ID Cohen M Maffulli N. Arthroscopic-Assisted broström-gould for chronic ankle instability: a long-term follow-up. Am J Sports Med. (2011) 39(11):2381–8. 10.1177/0363546511416069 21803979

[17] Karlsson J Bergsten T Lansinger O Peterson L. Reconstruction of the lateral ligaments of the ankle for chronic lateral instability. J Bone Joint Surg Am. (1988) 70(4):581–8. 10.2106/00004623-198870040-00015 3356725

[18] Brodsky AR O'Malley MJ Bohne WH Deland JA Kennedy JG. An analysis of outcome measures following the broström-gould procedure for chronic lateral ankle instability. Foot Ankle Int. (2005) 26(10):816–9. 10.1177/107110070502601005 16221453

[19] Buerer Y Winkler M Burn A Chopra S Crevoisier X. Evaluation of a modified broström-gould procedure for treatment of chronic lateral ankle instability: a retrospective study with critical analysis of outcome scoring. Foot Ankle Surg. (2013) 19(1):36–41. 10.1016/j.fas.2012.10.005 23337275

[20] Baumbach SF Braunstein M Herterich V Böcker W Waizy H Polzer H. Arthroscopic repair of chronic lateral ankle instability. Oper Orthop Traumatol. (2019) 31(3):201–10. 10.1007/s00064-019-0595-7 30918997

[21] Shahrulazua A Ariff Sukimin MS Tengku Muzaffar TM Yusof MI. Early functional outcome of a modified brostrom-gould surgery using bioabsorbable suture anchor for chronic lateral ankle instability. Singapore Med J. (2010) 51(3):235–41. PMID: 20428746

